# NALCN Channels Are Not Major targets of Gα _o_ or Gα _q_ Modulation in the *C. elegans* Egg-Laying Behavior Circuit

**DOI:** 10.17912/micropub.biology.001065

**Published:** 2024-01-11

**Authors:** Ariana Jose, Kevin Collins

**Affiliations:** 1 Physiology & Biophysics, University of Miami, Coral Gables, Florida, United States; 2 Biology, University of Miami, Coral Gables, Florida, United States

## Abstract

Sodium leak channels (NALCN) are regulators of cell membrane potential. Previous studies in mammalian neurons and *C. elegans* have shown that Gα _q_ and Gα _o_ signaling antagonistically modulates NALCN activity to regulate neuron excitability and neurotransmitter release for behavior. Here, we test whether NALCNs mediate the effects of Gα _q_ and/or Gα _o_ signaling in the *C. elegans* egg-laying circuit. We find that while gain-of-function NALCN mutants exhibit hyperactive egg-laying behavior, NALCNs are not required for the effects of Gα _q_ or Gα _o_ signaling for egg laying. These results show that NALCNs are not major effectors of G-protein signaling for *C. elegans* egg-laying behavior.

**Figure 1. NALCN channels are not required for Gα q , Gα o , serotonin, or DAG regulation of C. elegans egg-laying behavior.**
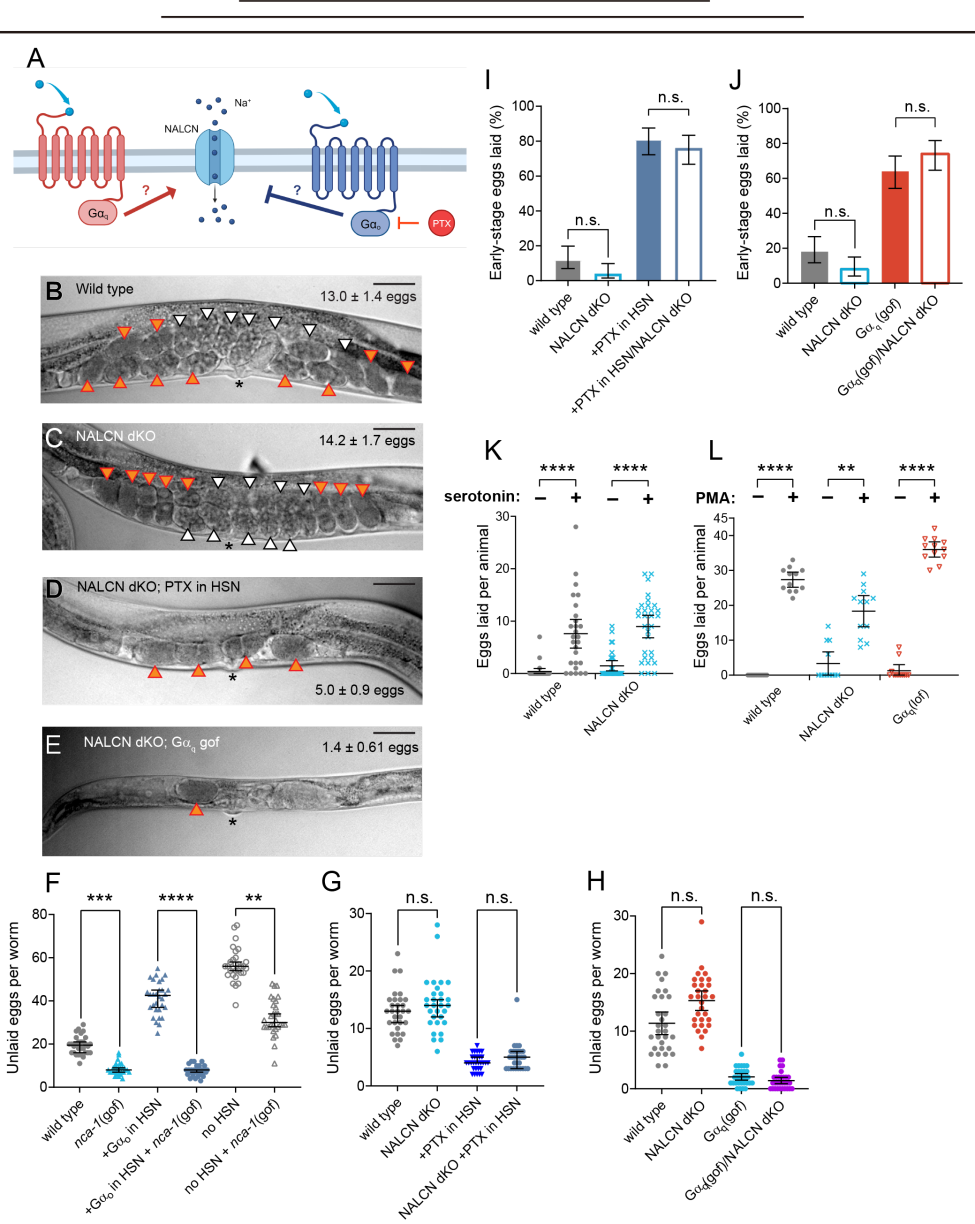
(A) Cartoon of G-protein signaling pathways through hypothesized NALCN modulation. Gα _q_ and Gα _o_ are activated by G-protein-coupled receptors (red or blue, respectively) and signal through either known or unknown effectors, respectively, to promote or inhibit cell excitability through ion channels, one of which may be NALCN. Pertussis Toxin (PTX) inhibits Gα _o_ signaling. (B-E) Micrographs of egg accumulation in wild-type (B) and mutant strains ZM2960 ( *nca-1( gk9 )* IV; *nca-2 ( gk5 )* III) (C), MIA524 ( *nca-1( gk9 )* IV; *nca-2 ( gk5 )* III; *vsIs50* [ *tph-1::* PTX, LIN-15 ] *lite-1 ( ce314 )* *lin-15 (n765ts)* X) (D), and EG4036 ( *egl-30 ( tg26 )* I*; *nca-1( gk9 )* IV; *nca-2 ( gk5 )* III) (E). Scale bars indicate 100 µm. *: denotes a gain-of-function allele of the Gα _q_ ortholog, *egl-30* . (F-H) Scatterplots of egg accumulation in wild-type and indicated mutant or transgenic animals with altered NALCN function, G-protein signaling, and/or HSN development. Error bars represent 95% confidence intervals for the mean. n.s. indicates P > 0.05; ** indicates P = 0.0036; *** indicates P = 0.0005; **** indicates P < 0.0001 (Kruskall-Wallis test with Dunn’s correction for multiple comparisons; n = 30 per genotype). (F) Animals expressing the *nca-1( e625 )* gain-of-function mutant along with either a gain-of-function Gα _o_ (Q205L) mutant transgenically expressed in the HSNs or a whole-animal *egl-1 ( n986dm )* mutation that drives HSN programmed cell death (e.g. no HSNs). (G) Egg accumulation in *nca-1* ; *nca-2* double mutants (NALCN dKO) alone, in animals expressing Pertussis Toxin in the HSNs, or (H) *egl-30 ( tg26 )* Gα _q_ gain-of-function mutants. (I-J) Bar graphs indicate percent of embryos laid at early stages of development (8 cells per embryo or less). Error bars indicate 95% confidence intervals for the mean proportion. n.s. indicates P > 0.05 (Fisher’s exact test; n = 100 embryos per genotype). (K) Scatterplots indicating the number of eggs laid per worm in two hours in M9 buffer alone or M9 plus 18.5 mM serotonin. Error bars indicate 95% confidence intervals for the mean. **** indicates P < 0.0001 (Kruskall-Wallis test with Dunn’s correction for multiple comparisons; n ≥ 26 animals per condition). (L) Scatterplots indicating the number of eggs laid per worm in two hours in M9 buffer alone or M9 plus 10 μM PMA. Error bars indicate 95% confidence intervals for the mean. ** indicates P = 0.0071, and **** indicates P < 0.0001 (Kruskall-Wallis test with Dunn’s correction for multiple comparisons; n = 12 animals for each indicated group).

## Description

Signaling through alpha subunits of heterotrimeric G-proteins such as Gα _q_ and Gα _o_ regulates cell electrical excitability and neurotransmitter release, yet which downstream ion channel effectors mediate their signaling *in vivo* is not well understood. *C. elegans* is an ideal system for the study of these pathways as its simple nervous system is fully mapped by electron microscopy, uses the same signaling molecules as in humans, and is rich in GPCRs homologous to those targeted therapeutically.

The egg-laying circuit of *C. elegans* consists, in part, of a pair of serotonergic hermaphrodite-specific neurons (HSNs), which innervate two pairs of vulval muscle cells that contract for egg release (Schafer, 2006) . Gα _q_ and Gα _o_ signal in both the HSNs and vulval muscles to promote and inhibit egg-laying behavior, respectively (Collins et al., 2016; Tanis et al., 2008) , and electrophysiological and Ca ^2+^ imaging experiments show that Gα _o_ signaling serves to stabilize the resting membrane potential of the HSNs, reducing their electrical excitability and Ca ^2+^ transient activity in the vulval muscles (Dhakal et al., 2022; Ravi et al., 2021; Shyn et al., 2003) .

Several studies in both mammals and worms have identified NALCN Na ^+^ leak channels as a potential effector of both Gα _q_ and Gα _o_ signaling ((Lu et al., 2009; Lutas et al., 2016; Philippart & Khaliq, 2018; Swayne et al., 2009; Topalidou et al., 2017a; Topalidou et al., 2017b); Fig. 1A ). In worms, double knockout mutants of NALCN genes *unc-77* (also known and referred to herein as *nca-1* ) and *nca-2* suppress the loopy locomotor waveform of either activated Gα _q_ *egl-30 ( tg26 )* mutants, animals expressing activated Rho, or loss-of-function Gα _o_ *goa-1* mutants (Topalidou et al., 2017a). Additionally, genetic experiments with several genes involved in the Gα _o_ and Gα _q_ -Rho pathways showed that dopamine signaling through Gα _o_ -coupled DOP-3 receptors negatively modulates NCA-1 and NCA-2 channels (Topalidou et al., 2017b). Whether NALCN channels play a similar role in other *C. elegans* behaviors including egg laying is not clear. *nca-1* and *nca-2* are expressed in cells of the egg-laying circuit (Taylor et al., 2021) , and gain-of-function *nca-1* mutations lead to increased HSN Ca ^2+^ activity (Yeh et al., 2008) and hyperactive egg-laying behavior (Yeh et al., 2008) ( Fig. 1A ), consistent with a role for NALCN channels in promoting egg laying.

To determine whether NALCN channels regulate egg laying downstream of G protein signaling, we used a genetic epistasis approach. Animals expressing the GTPase-deficient Gα _o_ mutant (Q205L) in the HSN neurons (Tanis et al., 2008) have reduced HSN Ca ^2+^ activity and delayed egg laying (Ravi et al., 2021) . Double mutants expressing both Gα _o_ (Q205L) and *nca-1(gf)* have strongly hyperactive egg-laying behavior that is not statistically different from *nca-1(gf)* mutant animals alone ( Fig. 1F ), consistent with NCA-1 channels acting downstream of Gα _o_ in egg laying. Depolarization in the egg-laying muscles can overcome defects in HSN function, including loss of HSNs altogether (Bastiani et al., 2003) . To test if the hyperactive egg laying of *nca-1* (gf) mutants represented HSN-specific suppression of Gα _o_ (Q205L) or a bypass, we created *nca-1(gf)* mutant animals lacking HSNs using the *egl-1 ( n986dm )* mutation that causes premature death of the HSN neurons (Conradt & Horvitz, 1998) . Unlike the results with Gα _o_ (Q205L), *nca-1(gf)* only partially suppressed the egg-laying defects caused by loss of HSNs, suggesting NCA-1 NALCN channels promote egg-laying behavior both in and outside of the HSNs.

To clarify the role of these channels more directly, we analyzed egg-laying behavior in *nca-1, nca-2* double knockout (dKO) mutants completely lacking NALCN channels. NALCN dKO animals retained a similar number of eggs in their uterus compared to wild-type animals (compare Fig. 1B to 1C; Fig. 1G ). We next tested how loss of NALCN channels affected egg-laying behavior in animals expressing Pertussis Toxin (PTX) in HSN to inactivate Gα _o_ (Tanis et al., 2008) or in *egl-30 ( tg26 )* gain-of-function mutants with a whole-animal increase in Gα _q_ signaling (Doi & Iwasaki, 2002) . We measured steady-state egg accumulation in each mutant and found that complete loss of NALCN function failed to suppress the hyperactive egg-laying behavior of either mutant ( Fig. 1D -E, G-H). Since mutants like *egl-30 ( tg26 )* grow slowly and have reduced brood sizes (Bastiani et al., 2003; Williams et al., 2007) , we complemented these experiments with assays of the developmental stage of freshly-laid eggs (Chase & Koelle, 2004) , a measure less dependent on brood size and animal growth rates ( Fig. 1I -J). We found that loss of NALCN channels did not suppress the laying of early stage eggs by the *egl-30 ( tg26 )* gain-of-function mutant. Together, these results show that NALCN channels are not required for the increased egg laying in mutants with too much excitatory Gα _q_ or lacking inhibitory Gα _o_ signaling.

Egg laying in NALCN dKO mutants was similarly responsive to exogenous serotonin and phorbol esters. Worms placed in hypertonic M9 buffer are inhibited for egg laying, and this is restored in M9 buffer containing serotonin. NALCN dKO mutants laid eggs in response to exogenous serotonin at a rate comparable to that of the wild type ( Fig. 1K ). NALCN dKO mutants also showed a robust egg-laying response to exogenous phorbol-12-myristate-13-acetate (PMA), an analog of diacylglycerol (DAG) – one of two second messengers produced by the Gα _q_ signaling pathway ( Fig. 1L ). Taken together, these experiments indicate that while elevated NALCN channel activity can promote egg-laying behavior, NALCN channels are not major effector targets of either Gα _q_ or Gα _o_ signaling for egg laying.

## Methods

**Strains**

All *C. elegans* strains, derived from the N2 wild-type strain, were cultured on a lawn of *E. coli* strain OP50 on nematode growth media (NGM) agar plates as previously described (Brenner, 1974) . The following strains were provided by the Caenorhabditis Genetics Center: N2 (wild type), VC12 *nca-1( gk9 )* *IV* (Consortium, 2012), VC9 *nca-2 ( gk5 )* *III* (Consortium, 2012), ZM2960 *nca-1( gk9 ) IV* ; *nca-2 ( gk5 )* *III* (Humphrey et al., 2007) , DR1089 *nca-1( e625 )* *IV* (Yeh et al., 2008) , MT1434 *egl-30 ( n686 )* I (Trent et al., 1983) , and CG21 *egl-30 ( tg26 )* *I* ; *him-5 ( e1490 )* *V* (Garcia et al., 2001) . LX1832 *lite-1 ( ce314 )* X *lin-15 (n765ts)* *X* was a generous gift from Dr. Michael Koelle and has been previously described (Collins et al., 2016) . EG4036 *egl-30 ( tg26 )* *I* ; *nca-1( gk9 )* *IV* ; *nca-2 ( gk5 )* *III* was a generous gift from Dr. Erik Jorgensen and has been previously described (Topalidou et al., 2017a).

Strain MIA27 *egl-1 ( n986dm ) V* in which the HSNs undergo programmed cell death was generated as previously described (Garcia & Collins, 2019) . Strains MIA244 *vsIs49* *V* ; *lite-1 ( ce314 )* X *lin-15 (n765ts)* X expressing activated GOA-1 (Q205L) in the HSNs from the *tph-1* promoter and MIA218 *vsIs50* X; *lite-1 ( ce314 )* X; *lin-15 (n765ts)* X expressing Pertussis Toxin (PTX) in the HSNs from the *tph-1* promoter have been described previously (Ravi et al., 2021) . The following strains were newly generated in this study:

MIA469 : *vsIs49* V; *nca-1( e625 )* IV; *lite-1 ( ce314 )* *lin-15 (n765ts)* X

MIA496 : *nca-1( gk9 )* IV; *lite-1 ( ce314 )* *lin-15 (n765ts)* X

MIA497 : *nca-2 ( gk5 )* III; *lite-1 ( ce314 )* *lin-15 (n765ts)* X

MIA506 : *nca-1( gk9 )* IV; *vsIs50 lite-1 ( ce314 )* *lin-15 (n765ts)* X

MIA507 : *nca-2 ( gk5 )* III; *vsIs50 lite-1 ( ce314 )* *lin-15 (n765ts)* X

MIA508 : *egl-1 ( n986dm )* V; *nca-1( e625 )* IV

MIA524 : *nca-1( gk9 )* IV; *nca-2 ( gk5 )* III; *vsIs50 lite-1 ( ce314 )* *lin-15 (n765ts)* X

MIA525 : *nca-1( gk9 )* IV; *nca-2 ( gk5 )* III; *lite-1 ( ce314 )* *lin-15 (n765ts)* X

**Behavior Assays**

Egg retention in the uterus and early developmental stages of laid eggs were measured as previously described (Chase & Koelle, 2004) . Both assays were conducted with adult animals staged approximately 30 h after the late L4 stage.

**Pharmacological Assays**

Egg laying in response to either exogenous serotonin or PMA was measured as previously described (Banerjee et al., 2017; Dhakal et al., 2022; Kopchock et al., 2021) . Briefly, adult animals 30 h after the late L4 stage were placed in individual wells containing 100 μL of either M9 buffer (a hypertonic solution that normally inhibits egg laying) or either 18.5 mM serotonin (creatinine sulfate monohydrate salt, Sigma-Aldrich # H7752) or 10 μM PMA (Phorbol-12-myristate-13-acetate, Calbiochem # 524400) in a 96-well microtiter dish. The number of eggs and/or L1 larvae released in each well were counted after 2 h.

**Statistical Procedures**

Mean numbers of eggs retained in the uterus or of eggs laid in pharmacological assays were compared between genotypes via Kruskall-Wallis test with Dunn’s correction for multiple comparisons. Mean proportions of early-stage eggs laid were compared by Fisher’s exact test. All P values were calculated in GraphPad Prism version 9.2.0.

**Microscopy**

Animals were immobilized at 30 hours post-L4 with M9 buffer as previously described (Moresco, 2005) and images of eggs in the uterus taken using a Zeiss Axio Observer Z1 microscope **.**
